# Association between *CYP17A1* and *HSD3B1* gene polymorphisms and testosterone levels in Nigerian prostate cancer patients

**DOI:** 10.1038/s41598-025-18752-x

**Published:** 2025-09-29

**Authors:** Christogonus Chichebe Ekenwaneze, Suleiman Zakari, Emmanuel Chimuebuka Amadi, Mary Okesola, Solomon Oladapo Rotimi, Ademola Oyekan, Olamijulo Fatiregun, Emeka Eze Joshua Iweala, Folakemi Odedina, Olubanke Olujoke Ogunlana

**Affiliations:** 1 Department of Biochemistry, College of Science and Technology, Covenant University, KM. 10 Idiroko Road, Canaan Land, Ota, 112104 Ogun State Nigeria; 2 Covenant Applied Informatics and Communication Africa Centre of Excellence (CApIC-ACE), Covenant University, Ota, Nigeria; 3 Cancer Research Group, Department of Biochemistry, College of Medicine, Federal University of Health Sciences, Otukpo, Benue State Nigeria; 4 Lagos State University Teaching Hospital (LASUTH), Ikeja, Lagos State Nigeria; 5 Lagos University Teaching Hospital (LUTH), Surulere, Lagos State Nigeria; 6 Community Outreach and Engagement Office and Programs, Mayo Clinic Comprehensive Cancer Centre, 4500 San Pablo Road, Jacksonville, FL 3224 USA

**Keywords:** Prostate cancer risk, Single nucleotide polymorphism, Androgen metabolism, Molecular biomarker, Hormonal regulation, Cancer, Cancer genetics, Cancer genomics

## Abstract

Prostate cancer (PCa) is a primary global health concern and the leading cause of cancer-related deaths in men. Genetic variation in androgen pathways is essential in PCa development and progression. Cytochrome P450 17A1 (*CYP17A1*) gene encodes a critical metabolic enzyme involved in testosterone (TT) synthesis, as it converts cholesterol into androstenedione. Similarly, the 3β-hydroxysteroid dehydrogenase type 1 (*HSD3B1*) gene encodes an enzyme that catalyses the conversion of dehydroepiandrosterone (DHEA) to androstenedione, a critical precursor for TT production. The case-control study was conducted on 40 PCa patients and 40 healthy males with matching ages. Detection of *CYP17A1* and *HSD3B1* polymorphisms was done using the TaqMan genotyping assay, and estimation of TT levels in serum was done using the enzyme-linked immunosorbent assay technique. Detected genotypes were AA, AG, and GG for *CYP17A1*, and AA and CA for *HSD3B1*; the adrenal-permissive CC genotype of *HSD3B1* was absent. The TT levels were significantly lower in PCa patients (*p* = 0.00148). No significant associations were found between polymorphisms in *CYP17A1*, *HSD3B1* and TT levels. The HSD3B1 CA genotype showed a non-significant trend toward increased PCa risk (OR = 2.39, *p* = 0.183) that requires validation in larger studies before any clinical relevance can be established.

## Introduction

Prostate cancer (PCa) is the most common hormone-driven cancer in men worldwide and the second most prevalent among Nigerian men^1,2^. Its development involves complex interactions between genetic and environmental factors, with androgens playing a central role in disease onset and progression^3^. The PCa cells rely on androgens like testosterone (TT) and dihydrotestosterone (DHT) to activate the androgen receptor (AR), which regulates genes involved in cell growth, survival, and migration^4,5^.

Androgen biosynthesis occurs via classical, alternative, and backdoor pathways^6^. Two key enzymes in this process, CYP17A1 and HSD3B1, are encoded by genes implicated in androgen regulation. The *CYP17A1* catalyses a critical step in converting cholesterol to androstenedione, while *HSD3B1* converts DHEA into androstenedione, a TT precursor^6,7^. Genetic variants in these genes may alter enzyme activity, influencing androgen levels and, consequently, PCa behaviour^8^.

Scientists have found that PCa can behave differently depending on a person’s racial background^9^. Evidence suggests that genetic polymorphisms, such as CYP17A1 rs743572 and HSD3B1 rs1047303 (1245 A→C), can impact androgen synthesis and resistance to androgen deprivation therapy (ADT), potentially contributing to treatment failure in advanced PCa^10,11,7^. The C allele in HSD3B1 leads to a more stable enzyme, promoting continuous androgen production even under ADT, a key mechanism in castration-resistant PCa (mCRPC)^12^. The ADT stands as a common approach to treating PCa. However, many patients eventually develop resistance to ADT, suggesting that other factors, such as genetic variations in specific androgen receptor genes, such as CYP17A1 and HSD3B1, along with their expression levels, may play a role in the progression of PCa^13^.

Studies on these polymorphisms have shown varied outcomes across populations, especially in Caucasian and Asian men, but data remain scarce in African cohorts. Racial differences in PCa progression and response to therapy underscore the importance of region-specific research^9,14^. However, previous studies link these SNPs to TT levels and PCa outcomes^15,7^. None have explicitly focused on Nigerian men, despite the high disease burden and mortality. The study investigated *CYP17A1* rs743572 and HSD3β1 rs1047303 gene polymorphisms and testosterone levels in Nigerian men with PCa. Given the role of these genes in androgen metabolism and potential influence on ADT response, the findings may offer insight into PCa biology in African populations and guide future precision oncology efforts.

## Materials and methodology

### Participants

The study used a case-control design of eighty (80) men between the ages of 45 and 85. It included prostate cancer patients, confirmed through histological diagnosis, from Lagos University Teaching Hospital (LUTH) and Lagos State University Teaching Hospital (LASUTH), along with healthy men. Participants were divided into two groups: Group I consisted of forty men with PCa, while the control group included forty healthy men of similar ages.

All measures were carried out following the ethical considerations of the Covenant Health Research Ethics Committee (CHREC), Covenant University P.M.B. 1023, Ota, Ogun State, Nigeria (approval number ORG0010037), as well as the ethical standards of the 1964 Declaration of Helsinki. All participants provided informed consent before data collection and following the explanation of research objectives.

### Specimen collection and DNA extraction

Venous blood samples (5 ml) were collected from each subject under aseptic conditions using plain vacutainer tubes and divided into two aliquots. One aliquot, containing 3 ml, was transferred into sterile EDTA vacutainer tubes, mixed thoroughly, and stored at −20 °C for DNA extraction. The other aliquot, 2 ml, was left to clot, centrifuged at 1000×g for 10 min, and the sera were separated and stored at −20 °C for testosterone analysis.

The extraction of genomic DNA from peripheral blood leucocytes of EDTA anti-coagulated blood was performed using the DNA extraction kit Aidlab Blood and Tissue Mini Kit (Beijing, China). Before quantitative polymerase chain reaction (PCR) analysis, pure genomic DNA samples were measured using ultraviolet absorbance at 260 nm using a Thermo Scientific NanoDrop TM and kept at 20 °C.

### Genotyping of *CYP17A1* rs743572 A/G and HSD3β1 rs1047303 C/A polymorphisms

Two SNPS, *CYP17A1* rs743572 and HSD3β1 rs1047303, were selected for evaluation based on the NCBI dbSNP database (http://www.ncbi.nlm.nih.gov/projects/SNP) and publication. All PCRS were prepared in a volume of 10 µL, containing TaqMan Universal PCR Master Mix, specific TaqMan SNP Genotyping Assays (Applied Biosystems), nuclease-free water (Invitrogen/Life Technologies, USA), totalling 7 µL, and genomic DNA (3 µL). Thermal cycling conditions were 40 cycles of 10 min at 95 °C, 92 °C for 15 s, and 60 °C for 1 min. Genotyping of *CYP17A1* rs743572 and HSD3β1 rs1047303 SNPS was performed by TaqMan PCR using the TaqMan allelic discrimination system (QuantStudio.™ 5 Real-Time PCR System). All results were automatically called by TaqMan genotyping assay software version 1.7.1. The quality value for all genotype calls (with a 99% certainty) was calculated. From the 80 samples collected, a subset failed genotyping due to low DNA concentration and purity, related to sample degradation and suboptimal storage conditions before DNA extraction. Despite quality control steps, approximately 15–22.5% of samples yielded insufficient signal strength during TaqMan allelic discrimination.

### Quantification of testosterone by ELISA method

Quantitative assessment of serum testosterone was carried out using the enzyme-linked immunosorbent assay (ELISA) technique (ELISA Kit, Catalogue number: DS177714) supplied by Biovantion Inc., China. The test samples (human serum) and incubation buffer were first added to a pre-coated microplate well and shaken for 10 min to mix. A testosterone enzyme conjugate was added to the microplate wells and gently shaken for 30 s. The plates were then covered with a plate lid. Incubation was carried out at 37 °C for 60 min. Following incubation, decantation, and washing each microplate with wash solution five times, the substrate was added to each well with no shaking of the wells; the plate was incubated at ambient temperature (18–25 °C) in the dark for 20 min. The reaction was terminated with the addition of a stop solution to each well and shaked for 15–20 s. The colour was allowed to change completely, from blue to yellow, and absorbance was read at 450 nm using the ELISA reader.

### Data analysis

A Student’s *t*-test was used to compare the means of testosterone levels between the case and control groups. Genotypic and allelic distributions of CYP17A1 rs743572 and HSD3Β1 rs1047303 polymorphisms were compared using the Chi-square test. Multivariate logistic regression models were used to assess the association between prostate cancer (PCa) risk and genotype using a dominant model (AG + GG vs. AA for CYP17A1, and CA vs. AA for HSD3B1), while adjusting for testosterone level as a continuous covariate. Regression plots were generated in R (v4.4.1) using ggplot2, visualising predicted probabilities of PCa across testosterone levels, stratified by genotype. All statistical analyses were performed using R (version 4.4.1) and Microsoft Excel. All studied SNPS conformed to Hardy-Weinberg equilibrium in the control group. Odds ratios (OR) with 95% confidence intervals (CI) were calculated to assess the association between genetic variants and prostate cancer susceptibility. A *p*-value < 0.05 was considered statistically significant.

- Core facilities-Cancer Genomics Lab at Covenant University.
- Instruments-QuantStudio™ 5 Real-Time PCR System (Applied Biosystems), NanoDrop™ One UV-Vis Spectrophotometer (Thermo Scientific), ELISA Microplate Reader, Centrifuge.
- Online resources/databases-NCBI dbSNP (https://www.ncbi.nlm.nih.gov/snp).
- Organisms-Not applicable.
- Services
- Software tools-R (version 4.4.1), Microsoft Excel, QuantStudio™ Design & Analysis Software (v1.7.1).

## Results

**Fig. 1:**
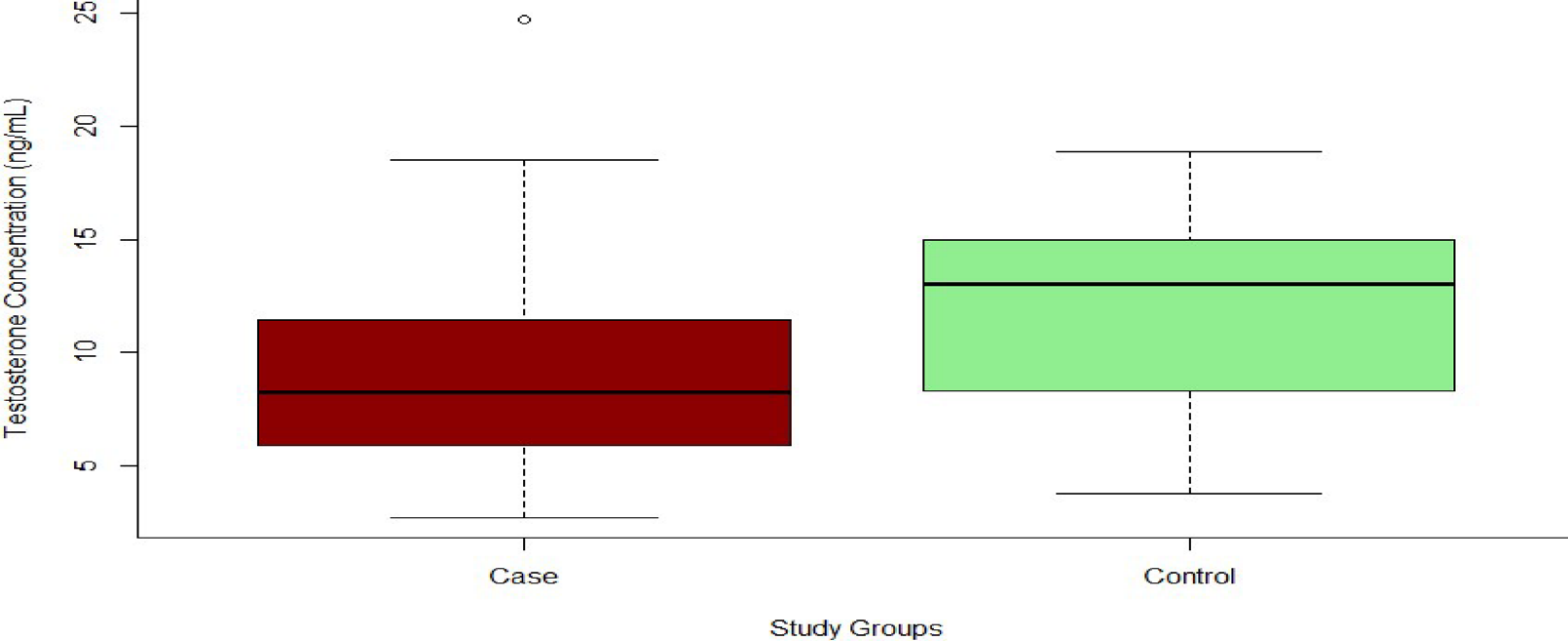
Box plot showing the distribution of serum testosterone levels in prostate cancer patients and healthy controls (Case = red, Control = Green). Median testosterone levels were significantly lower in the PCa group than in controls (*p* = 0.00148*). Boxes represent the interquartile range (IQR); whiskers indicate the full range. Asterisk (*) denotes statistical significance at *p* < 0.05.

**Fig. 2:**
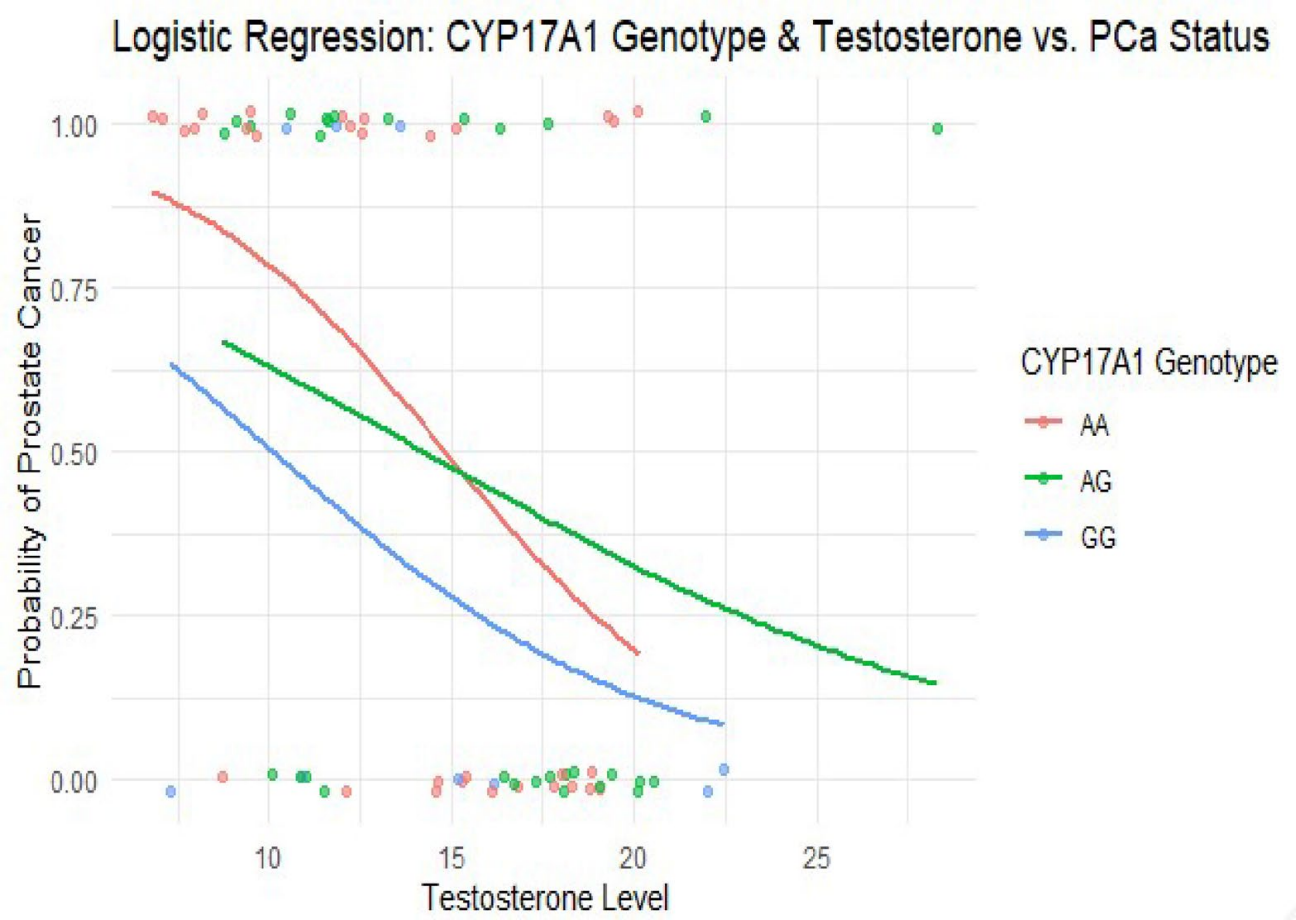
Regression curve illustrating CYP17A1, testosterone and PCa susceptibility. Predicted probabilities of prostate cancer across testosterone levels by CYP17A1 genotype (AA = red, AG = green, GG = blue); testosterone was significantly associated with risk (*p* = 0.003).

**Fig. 3:**
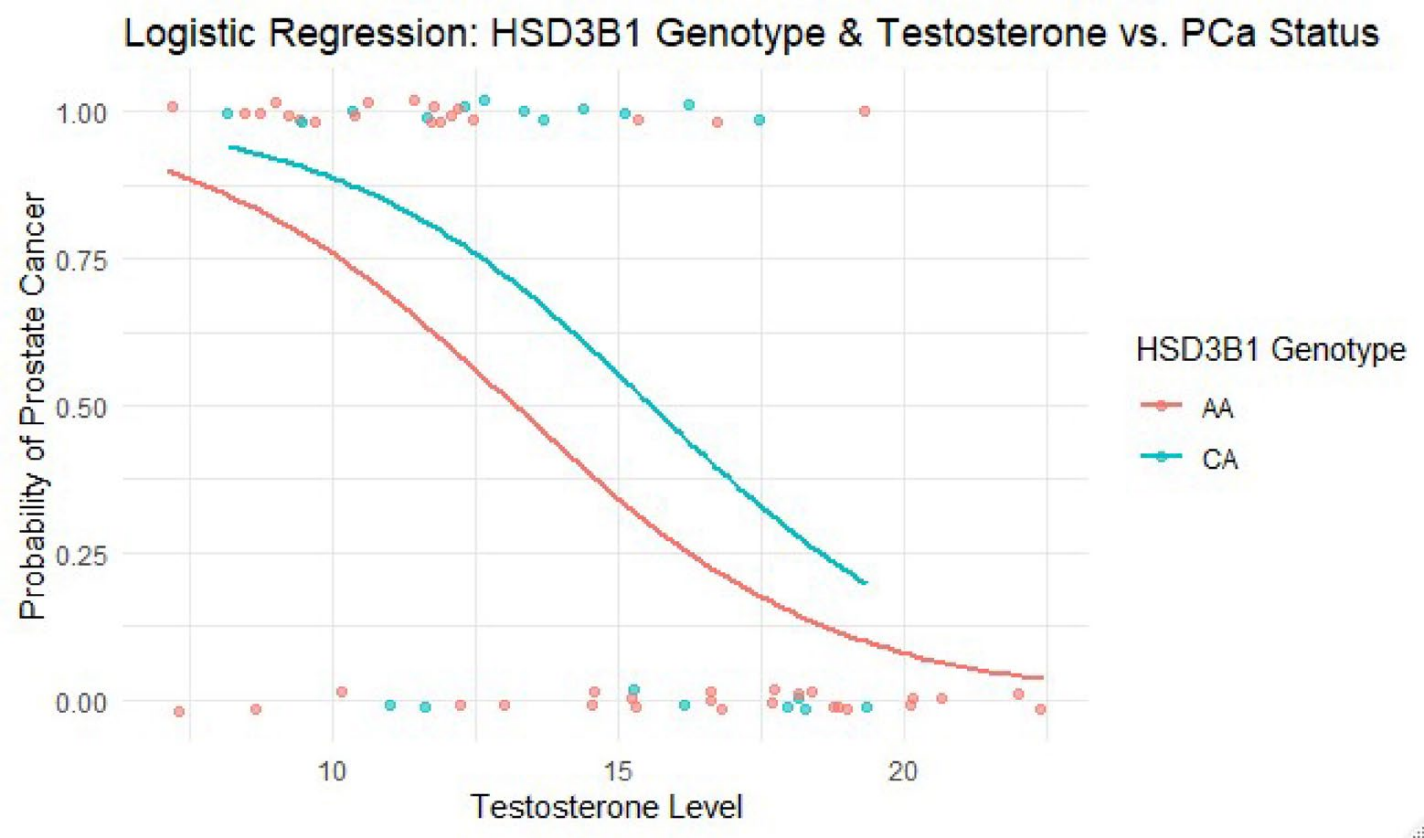
Regression curve showing HSD3Β1, testosterone, and PCa susceptibility. Predicted PCa probabilities by testosterone level and HSD3B1 genotype (AA = red, CA = teal); testosterone was significantly associated with risk (*p* = 0.001), while genotype was not.

Results of the present study are shown in Tables (1, 2, 3, 4, 5, 6) and Figs. (1, 2, 3).

### Descriptive data

Table 1 presents baseline characteristics of the prostate cancer group (*n* = 40). The average age was 70; most patients were from the Hausa ethnic group. About 32% had a tertiary education, and 11% had a family history of cancer. Over 70% had ECOG scores of 3 or 4, indicating advanced disease. All patients had adenocarcinoma and received anti-androgen therapy, with 75% also undergoing chemotherapy. Most PCa were diagnosed between the ages of 60 and 79, and over 55% had high-grade disease based on Gleason scores.

**Table 1:** Baseline clinical and prostate cancer Characteristics.

Characteristics		Values
Age	Average age	70 years
Weight	Average weight	66.46 kg
Height	Average height	157 cm
Educational level	None	17.86%
	Primary	28.57%
	Secondary	21.43%
	Tertiary	32.14%
Tribe in Nigeria	Benin	3.57%
	Ebira	3.57%
	Fulani	14.29%
	Hausa	42.86%
	Hausa	7.14%
	Igala	3.57%
	Igbo	7.14%
	Ora	3.57%
	Yoruba	14.29%
Family history of cancer	Yes	11.11%
	No	88.89%
ECOG score	1	20.83%
	2	8.33%
	3	37.50%
	4	33.33%
Chemotherapy	Yes	75%
	No	25%
Age of diagnosis	45–59	13.33%
	60–79	50%
	80 and above	10%
	N/A	26.67%
Histology type	Adenocarcinoma	100%
Anti-androgen treatment	Yes	100%
Gleason score	5	3.68%
	6	22.06%
	7	10.29%
	8	35.29%
	9	6.62%
	10	22.06%
Gleason grade	High grade	55.56%
	Intermediate grade	11.11%
	Low grade	33.33%

Data are represented as percentages, kilograms (kg), and centimetres (cm). Eastern cooperative oncology group (ECOG)

Table 2 shows the baseline characteristics of the control group (*n* = 40). The average age was 51, notably younger than the case group (70 years). Controls also had a higher average weight (87.11 kg) than cases, while height was similar (155 cm), which may reflect general population differences rather than disease-specific traits.

**Table 2:** Baseline characteristics of control Group.

Characteristics		Values
Age	Average age	51 years
Weight	Average weight	87.11 kg
Height	Average height	155.00 cm

Data are represented as kilograms (kg) and centimetres (cm)

### Genotyping result for *CYP17A1*

As shown in Table 3, the distribution of *CYP17A1* genotypes (AA, AG, GG) and alleles (A, G) did not differ significantly between PCa cases and controls. The AA genotype was more frequent among cases (50.00%) compared to controls (38.89%), while the GG genotype was more common in controls (16.67%). However, these differences were insignificant (*p* = 0.5047 for genotype; *p* = 0.3161 for allele).

**Table 3:** Frequency of distribution of CYP17A1 allele and Genotype.

CYP17A1		Case	Control	OR	95%CI (Lower- Upper)	*p*_value
Genotype	AA	17/33 (50.00%)	14/37 (38.89%)	2.07	0.47–9.02	0.5047
	AG	14/33 (41.18%	16/37 (44.44%)			
	GG	3/33 (8.82%)	6/37 (16.67%)			
Allele	A	48/68 (70.59%)	44/74 (61.11%)	1.53	0.75–3.09	0.3161
	G	20/68 (29.41%)	28/74 (38.89%)			

Data are represented as frequency and percentage, OR (Odds ratio), and CI (Confidence interval)

### Genotyping result for *HSD3Β1*

In Table 4, the HSD3B1 CA genotype was more frequent among PCa cases (38.71%) than controls (25.00%), suggesting a possible increased risk (OR = 1.89). However, this association was not statistically significant (*p* = 0.3692). The adrenal-permissive CC genotype was absent in both groups. At the allelic level, the C allele was slightly more prevalent in cases (19.35%) than controls (13.33%), but this difference was also not significant (*p* = 0.5134).

**Table 4:** Frequency distribution of HSD31 allele and Genotype.

HSD3Β1		Case	Control	OR	95%CI (lower-upper)	*p*_value
Genotype	AA	19/31 (61.29%)	24/32 (75.00%)	1.89	0.75–4.74	0.3692
	CA	12/31 (38.71%)	8/32 (25.00%)			
	CC	N/A	N/A	N/A	N/A	N/A
Allele	A	50/62 (80.65%)	52/64 (86.67%)	1.56	0.55–4.40	0.5134
	C	12/62 (19.35%)	8/64 (13.33%)			

Data are represented as frequency and percentage, OR (Odds ratio), N/A (Not applicable), and CI (Confidence interval)

### Levels of testosterone

Testosterone levels were significantly lower in PCa patients compared to controls (*p* = 0.00148) in Fig. 1; however, all prostate cancer patients were receiving anti-androgen therapy, which is known to suppress testosterone levels.

### Association of *CYP17A1* and *HSD3Β1* genotypes with testosterone

Table 5 presents multivariate logistic regression results for the association between CYP17A1 genotypes, testosterone levels, and PCa risk. Compared to the reference AA genotype, AG and GG showed no significant associations with PCa risk (*p* = 0.861 and 0.266, respectively). However, testosterone levels were inversely associated with PCa risk (OR = 0.83; *p* = 0.003).

**Table 5:** Multivariate logistic regression results for CYP17A1 genotypes, testosterone and prostate cancer Risk.

	Odds ratio (OR)	95% CI (lower–upper)	*p*-Value
CYP17A1 AA (Intercept)	17.46	2.45–124.66	0.004
CYP17A1 AG vs. AA	0.91	0.30–2.70	0.861
CYP17A1 GG vs. AA	0.39	0.07–2.07	0.266
Testosterone	0.83	0.73–0.94	0.003*

CI , Confidence Interval; OR , Odds Ratio. Genotype AA serves as the reference group

* indicates statistical significance at *p* < 0.05

Figure 2 Multivariate logistic regression curves show the predicted PCa probability across serum testosterone levels, stratified by *CYP17A1* rs743572 genotypes (AA, AG, GG). Across all genotypes, a decrease in testosterone levels is associated with an increased probability of PCa. The association was statistically significant for testosterone (*p* = 0.003), while genotype differences were not. Each curve represents the fitted probability line per genotype; colored dots indicate individual observations.

As shown in Table 6, testosterone levels were significantly associated with the predicted risk of PCa (OR = 0.70; *p* = 0.001*). The HSD3B1 CA genotype showed a non-significant trend toward increased risk (OR = 2.39; *p* = 0.183) compared to the AA reference group.

**Table 6:** Multivariate logistic regression results for HSD31 genotypes, testosterone and prostate cancer Risks.

Predictors	Odds ratio (OR)	95% CI (lower–upper)	*p*-value
HSD3Β1 AA (Intercept)	118.54	8.86–1586.30	0.0003
HSD3Β1 CA vs. AA	2.39	0.66–8.61	0.183
Testosterone	0.70	0.58–0.84	0.001*

CI, Confidence Interval; OR , Odds Ratio. Genotype AA serves as the reference group

Asterisk (*) indicates statistical significance at *p* < 0.05

Figure 3 Logistic regression curve showing the predicted probability of PCa across serum testosterone levels, stratified by *HSD3B1* rs1047303 genotypes (AA and CA). Lower testosterone levels were significantly associated with increased PCa risk (*p* = 0.001), while the CA genotype showed a non-significant trend toward higher risk (*p* = 0.183).

## Discussion

Prostate cancer (PCa) is a clinically and genetically heterogeneous disease^16^. The usual diagnosis methods, such as PSA level, core biopsies, T stage, and Gleason scores, offer limited information to determine the diagnosis and treatment options for the disease. The current clinical methods classify patients wrongly, leading to overtreatment or undertreatment of these diseases^17^. As precision medicine advances, genetic biomarkers have gained attention for their potential to enhance PCa risk stratification and treatment decisions^18,16^. This study investigates the potential role of CYP17A1 (rs743572) and HSD3Β1 (rs1047303) polymorphisms and their association with testosterone levels in Nigerian men with PCa. The study found no significant association between CYP17A1 polymorphisms and PCa risk, aligning with previous studies from Caucasian and Korean populations, where no significant difference in CYP17A1 genotype distribution was observed^19,12^. However, conflicting data from Japanese and African American populations suggest that population-specific effects may exist^20^, highlighting the need for larger, multi-ethnic investigations in African populations.

The absence of HSD3Β1 CC genotype in the PCa and control groups is a significant finding of the study. This CC genotype is associated with aggressive PCa, poor response to androgen deprivation therapy (ADT), and worse mortality outcome^7,11^. Its absence in the study suggests a potential racial genetic variation in PCa progression among Nigerian men. The CA genotype appeared more frequently in PCa cases than controls; this difference was not statistically significant (*p* = 0.183) and should not be interpreted as a definitive risk marker. However, the elevated odds ratio may indicate a biological trend worth exploring in larger studies, particularly given the CA variant’s potential role in altered androgen metabolism. Previous research has suggested that the HSD3Β1 CA genotype is linked with altered androgen metabolism, which may help explain its higher prevalence in patients with PCa^21,22^. Several studies have reported racial differences in the distribution of the HSD3B1 CC genotype and its influence on PCa outcomes. A study of 1,567 Black men found that only 19 (1.2%) carried the CC genotype, in contrast to 383 (10.3%) of white patients^7^. The lower frequency of the CC genotype in Black men may partially explain their better response to abiraterone, an antiandrogen therapy, compared to white men. These studies with large sample sizes^21,22,7^, consistently report that PCa patients carrying the homozygous CC genotype have worse clinical outcomes, mainly because of its role in intratumoral androgen synthesis and resistance to ADT. Prizment et al. also confirmed that the gain-of-function allele in *HSD3Β1* rs1047303 is associated with PCa mortality in men with metastatic disease. This is because men with adrenal-permissive alleles (such as CC) exhibit higher resistance to ADT, allowing DHEA conversion into testosterone and DHT within the tumour microenvironment, sustaining cancer progression^10^.

The finding of significantly lower testosterone levels in PCa patients (*p* = 0.00148) is expected, given that all cases were undergoing ADT, which suppresses testosterone. However, the lack of pre-treatment hormone data prevents a meaningful assessment of whether low testosterone levels preceded disease onset or were simply a result of therapy. This represents a key limitation. Some prior studies have shown no consistent association between testosterone and PCa risk, emphasising the need for longitudinal hormone monitoring to clarify causality^23,24^.

We also conducted multivariate logistic regression to assess the combined influence of genotype and testosterone levels on PCa risk (Figs. 2 and 3). Neither CYP17A1 nor HSD3B1 showed statistically significant associations in these models, although testosterone remained an independent predictor of risk. The HSD3B1 CA genotype showed a non-significant trend toward increased PCa risk (OR = 2.39, *p* = 0.183, 95% CI: 0.66–8.61) that requires validation in larger studies before any clinical relevance can be established. However, other studies have shown that HSD3Β1 polymorphisms may influence PCa progression but not necessarily disease onset^21,22^. The increased odds ratio for HSD3Β1 CA in our study suggests that this genotype may still play a role in PCa susceptibility, possibly through its influence on androgen metabolism and intratumoral testosterone biosynthesis^10^. Importantly, our study did not examine genotype-treatment interactions, such as whether specific genotypes affect response to ADT. This analysis could have offered valuable clinical insights and should be considered in future studies. To our knowledge, we are the first to investigate CYP17A1 (rs743572) and HSD3Β1 (rs1047303) polymorphisms and their association with testosterone levels in Nigerian men with PCa. Although most of our results were statistically insignificant, they contribute novel insights into PCa genetics in Nigerian men. Notably, the study avoids overstating the clinical relevance of preliminary trends, instead positioning these findings as a foundation for future research. The absence of the HSD3B1 CC genotype and the suggestive trend of CA enrichment in cases warrant further investigation in larger, ethnically diverse cohorts.

This study is limited primarily by its small sample size, which restricts statistical power and increases the risk of Type II error. The genotyping completion rate was approximately 80–85%, possibly due to DNA quality issues. The missing genotype data may also introduce bias. Another key limitation of our study is the lack of pre-treatment testosterone measurements. Since all PCa patients were undergoing anti-androgen therapy at the time of sampling, the observed reduction in testosterone may reflect treatment effects rather than intrinsic hormonal differences linked to disease risk. This limits our ability to draw causal inferences about testosterone’s role in prostate cancer aetiology. Furthermore, post-hoc power analysis using G*Power 3.1 revealed that with our sample size and the observed odds ratio of 2.39, the statistical power was approximately 31.5%, indicating limited ability to detect moderate effects. Despite its limitations, this study is the first to investigate CYP17A1 and HSD3B1 polymorphisms in a Nigerian prostate cancer cohort, revealing population-specific genetic features that may inform biomarker development and precision oncology. Larger, multi-centre studies incorporating treatment response data and pre-treatment hormone levels are needed to validate whether HSD3B1 CA or absence of CC has predictive value in the Nigerian population. As genomic precision oncology expands, incorporating diverse populations will be critical to ensure equitable and effective care.

## Data Availability

All data and materials used or generated in this study are available and may be provided on request by the corresponding author.

## Abbreivations

PCa: Prostate cancer
*CYP17A1*: Cytochrome P450 17A1
*HSD3B1*: 3β-hydroxysteroid dehydrogenase type 1
TT: Testosterone
DHEA: Dehydroepiandrosterone
RT-PCR: Real-time polymerase chain reaction
AR: Androgen receptor
SNPs: Single nucleotide polymorphisms
ELISA: Enzyme-linked immune-sorbent assay
DHT: Dihydrotestosterone
mCRPC: Metastatic castration-resistant prostate cancer
ADT: Androgen deprivation therapy
LUTH: Lagos university teaching hospital
LASUTH: Lagos state university teaching hospital
FMC: Federal medical centre
ECOG: Eastern cooperative oncology group
